# Identifying Small‐Displacement Stereotypical Behavior of Captive Slow Lorises Based on AnimalYOLO‐Bytetrack Network

**DOI:** 10.1002/ece3.72304

**Published:** 2025-10-10

**Authors:** Ziqi Yang, Yating Du, Peifeng Li, Tingting Leng, Shaoyun Ding, Yuxin Zhang, Zhenzhen Lin, Yilin Yang, Yumai Fan, Yan Zhang, Changjun Zeng, Meng Xie, Qingyong Ni

**Affiliations:** ^1^ Key Laboratory of Agricultural Bioinformatics, Ministry of Education Sichuan Agricultural University Chengdu China; ^2^ State Key Laboratory of Swine and Poultry Breeding Industry, College of Animal Science and Technology Sichuan Agricultural University Chengdu China; ^3^ College of Information Engineering Sichuan Agricultural University Ya'an China; ^4^ Dehong Wildlife Rescue Center Forestry Bureau of Dehong Prefecture Dehong China; ^5^ College of Life Science Sichuan Agricultural University Ya'an China

**Keywords:** abnormal behavior, animal welfare, Lorisidae primates, real‐time tracking

## Abstract

Wild animals rescued and kept in captivity often display abnormal behaviors, notably in the form of stereotypic behaviors. These behaviors, characterized by purposeless and repetitive actions, can significantly affect an animal's quality of life and socialization processes. Existing methods for identifying stereotypic behaviors predominantly focus on individual animals or on behaviors involving large displacements with extensive movements and distinct trajectories. However, research addressing scenarios involving multiple animals or small‐displacement stereotypic behaviors remains limited. This paper aims to address this gap by analyzing small‐displacement stereotypic behaviors in multiple animals, specifically focusing on captive slow lorises. As a preliminary exploration of this research area, we propose a novel method for analyzing small‐displacement stereotypic behavior utilizing automatic tracking and periodic changes in movement amplitude. We employ the AnimalYOLO‐Bytetrack multi‐target tracking network to monitor and quantify the movement amplitude of the slow loris, segment the time‐series data to identify the specific periods of stereotypic behaviors, and analyze periodicity using autocorrelation functions and Fourier transforms. The method developed in this study demonstrates significant efficacy, achieving a detection precision of 96.7%, a recall rate of 80.3%, and a mean Average Precision (mAP) value of 88.9%. These results surpass the performance metrics of most existing detection networks. Furthermore, the method exhibits an average prediction error of 0.33 for cycle time and 0.76 s for the duration of stereotypic behavior, highlighting its effectiveness in predicting the stereotypical behavior of Lorisidae primates.

## Graphical abstract

This study analyzes small‐displacement stereotypical behaviors of multiple captive slow lorises and proposes a new method that utilizes automatic tracking and the analysis of the cyclical changes in movement amplitude. By employing the AnimalYOLO‐Bytetrack multi‐target tracking network to monitor and quantify the movements of slow lorises, a detection accuracy of 96.7% and a recall rate of 80.3% were achieved, with average prediction errors of 0.33 and 0.76 s, respectively, effectively predicting the stereotypical behaviors of Lorisidae primates.

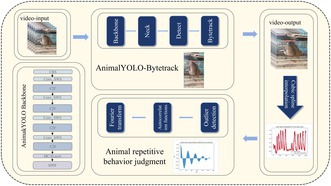

## 1 Introduction

The illegal wildlife trade remains a critical global threat, prompting increased confiscations and relocations of live animals by governmental organizations worldwide due to new legislation, stricter enforcement, and international bans on the pet trade (CTN News 2013; Crime 2021; Maddison 2019; Musing et al. 2016). Consequently, a significant number of rescued animals, including vulnerable species like the slow loris (Genus *Nycticebus*), spend their lives in captivity (Blair et al. 2021). Captive environments often induce chronic stress, leading to behavioral deterioration and the development of abnormal, repetitive behaviors known as stereotypies (Liu et al. 2021; Mathis et al. 2018; Schindler and Steinhage 2021; Wiltshire et al. 2023). These purposeless, invariant actions significantly impair an animal's physical health, psychological well‐being, social integration, and overall welfare, manifesting as growth retardation, reduced fertility, and loss of function (Rose et al. 2017). Stereotypies are constrained not only by physical space but also by the animal's perceived mental boundaries (Clubb and Mason 2007; Yin et al. 2024), and broadly fall into two categories: large‐displacement (e.g., pacing over large areas with visible trajectories) and small‐displacement (e.g., repetitive head‐shaking or circling within confined spaces).

The slow loris (Genus *Nycticebus*), a group of small, nocturnal, and arboreal primates within the family Lorisidae, is distinguished by its ultrasonic communication and venomous bite. Different *Nycticebus* species have varying International Union for Conservation of Nature (IUCN) Red List statuses ranging from Vulnerable (VU) to Critically Endangered (CR), with all species listed under Appendix S1 of the Convention on International Trade in Endangered Species (CITES). During rescue and rehabilitation, high captive pressure and facility shortages often necessitate housing in small cages, significantly increasing their susceptibility to stress and the development of pronounced stereotypical behaviors, even in accredited facilities (Grace et al. 2014). Common stereotypic behaviors in captive slow lorises include pacing, head bobbing, and circling, with small‐displacement movements like repetitive head shaking and tight circling being particularly prevalent (Moore et al. 2015). Effective observation and assessment of these behaviors are therefore crucial not only for ensuring the health and normal development of rescued individuals but also for informing effective conservation and management strategies, including improving captive conditions to mitigate stress and reduce stereotypy occurrence (Royle and Nichols 2003).

Traditional methods for monitoring slow loris behavior, relying on direct observation or short‐duration focal recordings, face significant limitations: they are labor‐intensive, time‐consuming, inefficient, prone to observer bias, and impractical for continuous, long‐term monitoring necessary to capture intermittent stereotypical episodes (Alejandro et al. 2021; Moore et al. 2015). While advances in deep learning and computer vision offer promising solutions for automated wildlife monitoring, existing automated approaches for behavior analysis often fall short in addressing the specific challenges posed by small‐displacement stereotypies and multi‐animal tracking. First, many studies focus primarily on detecting large‐displacement stereotypies by tracking significant positional changes or centroid trajectories (Yin et al. 2024), or concentrate on identifying behaviors in single animals (Feng et al. 2021), overlooking the complexities of multi‐individual settings. Second, methods relying on accelerometer data (Hathaway et al. 2023) or frame extraction for static posture classification (Marks et al. 2022) frequently fail to capture the temporal repetitiveness inherent in stereotypies over extended periods, lacking continuous periodicity analysis. Third, and most critically, research specifically addressing small‐displacement stereotypical behaviors—characterized by minimal movement amplitude and subtle trajectory changes, often occurring in multi‐animal environments with cluttered backgrounds like the stacked cages typical of rescue centers—remains severely limited (Clubb and Mason 2007). This limitation is especially problematic for species like the slow loris, whose cage‐confined stereotypic behaviors (e.g., head shaking, tight circling) exemplify these challenges. Compounding this gap, existing tracking algorithms struggle with occlusion, low‐confidence detections, and background clutter in such complex environments (Zhang et al. 2022).

This study develops and validates a novel, automated framework specifically designed for the continuous identification and analysis of small‐displacement stereotypical behaviors in captive animals, with a primary focus on multiple slow lorises. The core methodological approach integrates the AnimalYOLO‐Bytetrack multi‐target tracking network, the quantification of movement amplitude, and time‐series analysis of this amplitude signal. This integrated framework allows us to pinpoint not just the occurrence, but also the specific timing, duration, and periodicity of small‐displacement stereotypic episodes. Therefore, the primary objectives of this study are to: (1) develop and optimize the AnimalYOLO‐Bytetrack network for accurate and efficient multi‐target tracking of slow lorises within the challenging cluttered backgrounds typical of captive rescue environments; (2) establish and validate a novel method for quantifying small‐displacement movements based on the periodic variation in the aspect ratio of detected bounding boxes fitted to the animal's contour; (3) implement a robust periodicity analysis pipeline to automatically identify and characterize stereotypical behavior episodes from the derived movement amplitude time series; (4) comprehensively evaluate the performance and accuracy of the proposed integrated framework in detecting specific small‐displacement stereotypies in captive slow lorises, utilizing established metrics for detection and behavioral prediction; and (5) explore the generalizability of the approach by applying it to videos of other taxa (e.g., bears, primates, domestic cats) exhibiting small‐displacement stereotypical behaviors.

## 2 Materials and Methods

### 2.1 Dataset

#### 2.1.1 Data Collection

This study was conducted at the Wildlife Shelter and Rescue Centre located in Dehong Prefecture, Yunnan Province (24.38287° N, 98.45872° E). Situated on the China–Myanmar border, the center experiences a subtropical monsoon climate, with an average temperature of 19.5°C, annual rainfall of 1680 mm, and an altitude of 660 m. These animals are mainly kept in brick and steel mesh cages (31 × 38 × 34 cm in size) covered with color steel tiles. The research team, granted access to the facility, employed a Sony PXW‐X280 digital video camera with a resolution of 1980 × 1080 pixels for data collection. To ensure the diversity of the data, we conducted multiple video shootings at a distance of 1–3 m from the cages. Considering the nocturnal nature of the slow loris, filming was conducted both during the day and at night. To capture detailed nocturnal behavior, a red‐light flashlight was used for auxiliary illumination, based on the reflection of red‐orange light from the eyes of the slow loris, as noted by Nekaris et al. (2014). We systematically observed and filmed both long‐term and short‐term rescued Bengal slow lorises ( *Nycticebus bengalensis* ), excluding those disabled. Ultimately, we collected a dataset comprising 82 video files of 115 individuals (Figure S1).

#### 2.1.2 Target Detection Dataset

To improve the target detection model's accuracy for individual recognition and detection, we selected 69 out of 82 available video files (excluding those showing stereotypical behaviors) for training with substantial image data. The initial step in the process involved extracting frames from the video data at a rate of one frame per second. However, the nocturnal activity patterns of the slow loris, combined with the limitations inherent in nighttime filming, resulted in some frames being blurred, which posed challenges for accurately identifying individuals. After a meticulous selection process, 1829 clear and identifiable images were retained. These images were then annotated using LabelImg. To address the differences in pixel quality between daytime and nighttime images and to facilitate subsequent behavioral analysis, the annotations were categorized into two groups. To enhance the diversity of the dataset and improve the model's generalization capability, data augmentation techniques were applied. This process included randomly rotating the labeled dataset, adjusting image brightness, and adding noise (Figure S2). Consequently, the experimental dataset expanded to 7316 images. For the purposes of model training and performance evaluation, the dataset was divided into a training set, a validation set, and a testing set in a 7:2:1 ratio.

To illustrate the universality and effectiveness of our research method in identifying small displacement stereotypical behaviors across various animal species, we collected a diverse array of video materials from multiple web resources. In addition, we gathered a substantial number of animal images to process in consistency with the previously established target detection dataset for the slow loris. This initiative led to the development of a comprehensive target detection dataset that included polar bear, brown bear, primates (chimpanzee and rhesus macaque), and domestic cats, totaling 4441 images.

#### 2.1.3 Target Tracking Dataset

The objective of this paper is to conduct a comprehensive evaluation of the tracking algorithm's performance and to provide precise benchmark data for future methods of recognizing stereotypical behavior. To accomplish this, we processed 13 of the 82 videos in which the slow loris showed stereotypical behaviors, each frame in the video datasets has been meticulously labeled using Darklabel. These annotations thoroughly document the identification (ID) information of the animals within each video and accurately describe the coordinate position and size of the detection frame for each animal in every frame. A dataset specifically tailored for the stereotypical behavior of the slow loris was constructed and annotated. Furthermore, to assess the method's generalization capability, a separate dataset consisting of eight videos featuring stereotypical behaviors of various taxa (bears, primates, and domestic cats) was also collected and annotated (Figure S3).

### 2.2 Behavioral Tracking of Slow Loris Based on AnimalYOLO‐Bytetrack

**FIGURE 1:**
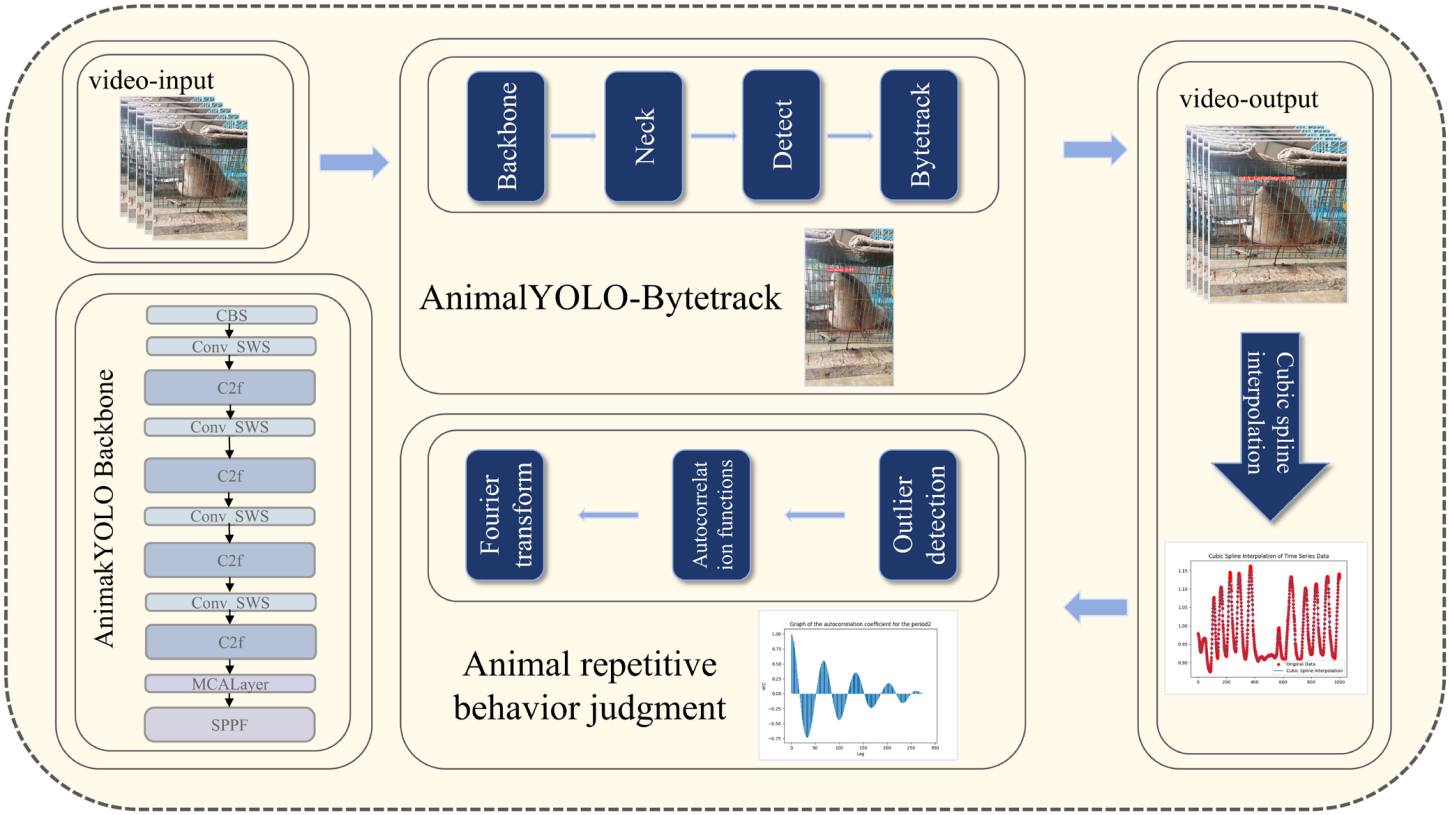
The network structure in identifying stereotypical behavior of slow lorises.

We designed a network structure, illustrated in Figure 1, to accurately analyze the behavior of the slow loris. Initially, we utilized the AnimalYOLO‐Bytetrack tracking algorithm to localize the individual and generate detection frames for each video sequence frame. To quantify the movement amplitude, we calculated the aspect ratio coefficients of the detection boxes for each frame. Using these coefficients, we then plotted movement amplitude change curves. These curves were subsequently segmented using an outlier point detection method to identify significant changes. To evaluate the periodicity of the movement amplitude changes, we plotted the autocorrelation function for each curve segment. For segments demonstrating clear periodicity, we conducted a Fourier transform to determine the period length.

#### 2.2.1 The Target Detection Modeling Framework

**FIGURE 2:**
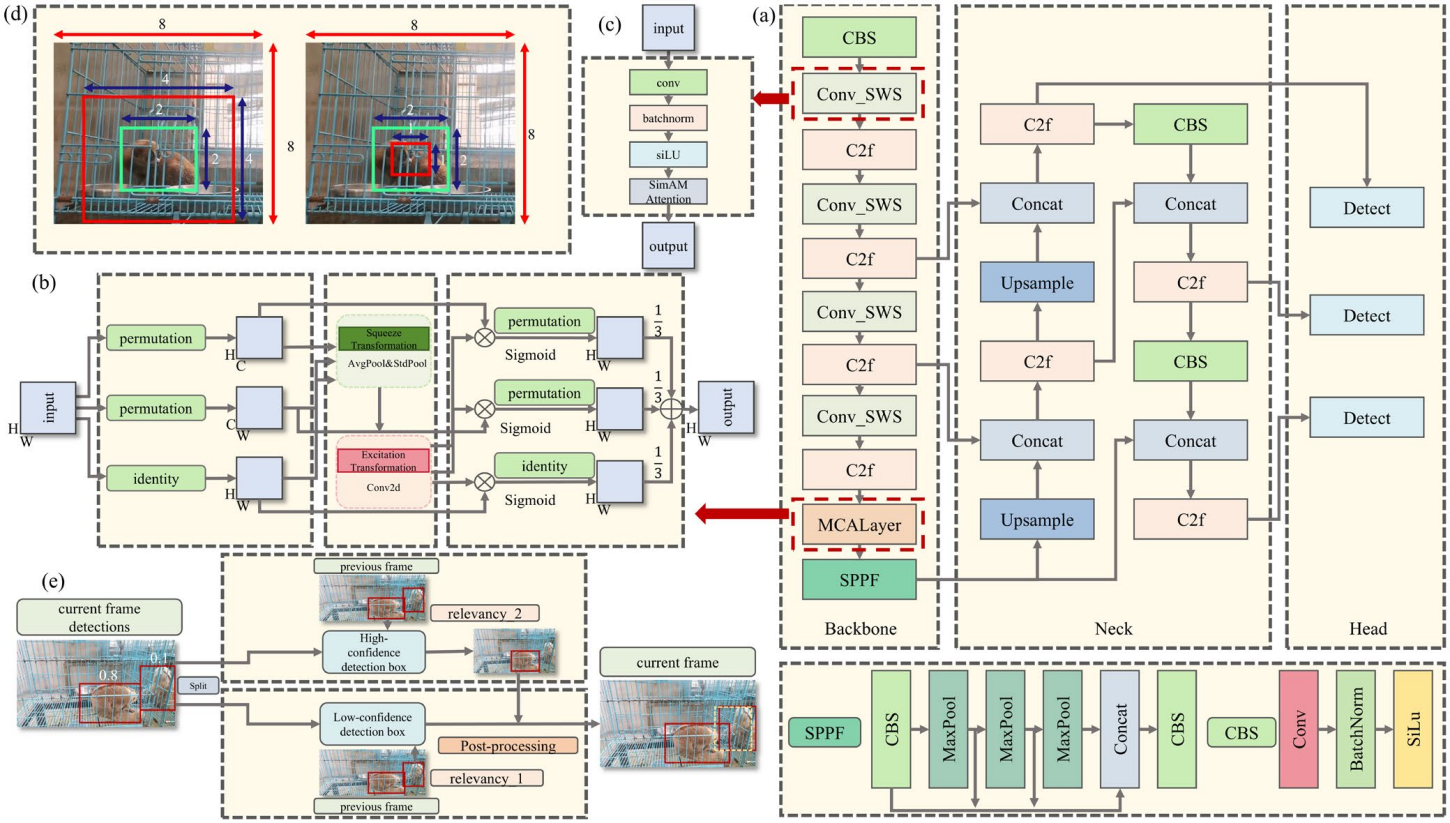
AnimalYOLO target tracking model structure. (a) The AnimalYOLO target detection model structure. (b) Multi‐dimension Cooperative Attention. (c) SWS Convolution Module. (d) MPDIoU algorithm. (e) ByteTrack tracking head.

The target detection framework in the AnimalYOLO‐Bytetrack model primarily builds upon the YOLOv8 algorithm, a member of the YOLO family. The network architecture consists of three key components: the Backbone, which extracts multi‐scale feature representations; the Neck, employing a PAN‐FPN structure to effectively fuse hierarchical features and enhance multi‐scale detection capability; and the Head, utilizing a decoupled design to separately output classification and regression predictions for improved detection accuracy. These components work synergistically to achieve efficient and robust target detection performance. The YOLOv8 algorithm enhances the backbone network's feature extraction capabilities by incorporating the SimAM Weighted Convolution Module (SWS) (Guo et al. 2025) and the Multidimensional Collaborative Attention (MCA) (Yu et al. 2023) (Figure 2a). Additionally, during the training process, it employs the latest Minimum Point Distance Intersection over Union (MPDIoU) (Ma and Xu 2023), which improves the alignment between the detection frame and the edge of the slow loris.

#### 2.2.2 Multi‐Dimension Cooperative Attention

In this study, the recognition of stereotypical behaviors was accomplished through the analysis of datasets obtained from videos recorded in complex environments. To improve recognition accuracy, the MCA mechanism was incorporated into the YOLOv8 backbone network, facilitating precise identification of the regions where slow lorises were situated. As illustrated in Figure 2b, the MCA mechanism comprised three parallel branches, each corresponding to the channel, height, and width dimensions. These branches independently captured feature dependencies specific to their respective dimensions, thereby enhancing feature representation through the application of attention weights.

Specifically, in the width branch, the input feature map undergoes a series of transformations. First, the feature map is rotated counterclockwise by 90° along the height (H) axis. The rotated feature map then passes through squeeze‐and‐excitation operations to derive the corresponding processed feature representation. Subsequently, attention weights for the width dimension are generated via a sigmoid activation function. These weights are applied through element‐wise multiplication with the rotated feature map to produce an enhanced feature representation. Finally, the processed feature map is rotated back by 90° to restore its original spatial orientation. The complete transformation process can be formally expressed as follows:

(1) $${F^{*}}_{W}=PM_{H}\left(F\right)$$

(2) F^W=TsqF*W,F~W=TexF^W

(3) $$A_{W}=\sigma \left({F^{\sim }}_{W}\right),F_{W}^{\prime }=A_{W}⨂{F^{*}}_{W},{F^{\prime \prime }}_{W}=PM_{H}^{-1}\left(F_{W}^{\prime }\right)$$

where *F* represents the original feature map, $PM_{H}$ involves rotating the feature map along the *H*‐axis. $T_{\mathrm{sq}}$ and $T_{\mathrm{ex}}$ correspond to applying squeeze and excitation transform operations respectively to the feature map. σ performs the sigmoid operation, followed by element‐wise multiplication in ⨂. Finally, ${F^{\prime \prime }}_{W}$ yields the resulting feature map after rotation.

The height branch follows a similar computational process as the width branch, with the key distinction being that when calculating attention weights for the height dimension, we utilize: (1) the feature map rotated 90° counterclockwise about the *W*‐axis, and (2) the excitation‐transformed feature map for sigmoid activation. The specific mathematical formulations are presented below:

(4) $${F^{*}}_{H}=PM_{W}\left(F\right)$$

(5) F^H=TsqF*H,F~H=TexF^H

(6) $$A_{H}=\sigma \left({F^{\sim }}_{H}\right),F_{H}^{\prime }=A_{H}⨂{F^{*}}_{H},{F^{\prime \prime }}_{H}=PM_{W}^{-1}\left(F_{H}^{\prime }\right)$$

$PM_{W}$ represents a rotation of the feature map along the *W*‐axis.

For the channel branch processing, the system first applies constant mapping to the input feature map. This is followed by sequential squeezing and excitation transformations. The transformed features then undergo sigmoid activation to generate channel‐wise attention weights, consistent with the other branches. These weights are element‐wise multiplied with the originally mapped feature maps. Finally, another constant mapping operation produces the output feature maps. The mathematical formulation is presented below:

(7) $${F^{*}}_{C}=\mathrm{IM}\left(F\right)$$

(8) F^C=TsqF*C,F~C=TexF^C

(9) $$A_{C}=\sigma \left({F^{\sim }}_{C}\right),F_{C}^{\prime }=A_{C}⨂{F^{*}}_{C},{F^{\prime \prime }}_{C}=\mathrm{IM}\left(F_{C}^{\prime }\right)$$

where IM denotes constant mapping processing.

Finally, in the integration stage, the outputs of the three branches are averaged and aggregated to produce the final refined feature map, as expressed by the following equation:

(10) $${F^{\prime \prime }}_{C}=\frac{1}{3}⨂\left({F^{\prime \prime }}_{W}⨁{F^{\prime \prime }}_{H}⨁{F^{\prime \prime }}_{C}\right)$$

In summary, the MCA module enables the model to simultaneously model attention across three dimensions: channel, height, and width. It effectively leverages the complementary relationships among these dimensions, allowing for more comprehensive feature association capture compared to focusing on a single dimension or treating channel and spatial dimensions separately. This enhances the richness and discriminative power of feature representations, making it particularly suitable for detecting slow loris targets in our cluttered background dataset.

#### 2.2.3 SWS Convolution Module

In wildlife rescue centers, slow lorises were often housed in environments with cluttered backgrounds, posing a substantial challenge for accurately identifying their location and extracting relevant feature information. To address this issue, this paper introduced a novel processing technique, the SWS convolution module, as depicted in Figure 2c. This module combined the SimAM attention mechanism with a convolutional layer, thereby enhancing the model's ability to emphasize key features while suppressing irrelevant ones.

Among them, the SimAM attention mechanism achieves fine‐grained local feature enhancement by partitioning the input feature map into four sub‐blocks and independently computing attention weights for each sub‐block. The specific formulation is as follows:

(11) $$y=\frac{{\left(x-u\right)}^{2}}{4\left(\frac{\sum {\left(x-u\right)}^{2}}{n}+\lambda \right)}+0.5$$

where *x* represents a sub‐block of the input feature map, *u* denotes the mean value of the sub‐block, and *n* equals the number of pixels in the sub‐block minus one (with a small constant added to prevent division by zero). The attention weights are then activated through a Sigmoid function and element‐wise multiplied with the original sub‐blocks to generate enhanced feature outputs. Finally, the processed results from all four sub‐blocks are concatenated to form the final feature map.

By incorporating the SWS module, the model demonstrates significant improvements in both target region identification and segmentation accuracy, consequently enhancing overall detection performance. Additionally, the SWS convolution module effectively reduced the impact of background noise by diminishing features in the background region, which allowed the model to focus more precisely on the target area. This capability not only bolstered the model's robustness but also underscored the module's suitability for slow loris target detection and tracking in complex background environments.

#### 2.2.4 MPDIoU Algorithm

Accurate localization of the slow loris is essential in the implementation of the stereotypical behavior recognition process, as it minimizes potential errors in quantifying movement amplitude. To enhance this accuracy, we employed the MPDIoU algorithm in place of the original loss function within the AnimalYOLO network. The algorithm, which calculates the minimum horizontal distance, accounts for overlapping areas, centroid distance, and deviations in width and height, thereby providing a more precise loss metric. This is particularly advantageous when distinguishing between bounding boxes with identical aspect ratios but differing in size or location. By directly calculating the distance between keypoints of the predicted and actual frames, the algorithm enables the predicted frames to more accurately conform to the edges of the slow loris, thus reducing errors in later movement metric quantification.

For computing MDPIoU, we first extract the coordinates of the top‐left corners $\left(x_{1}^{\mathrm{prd}},y_{1}^{\mathrm{prd}}\right)$ and $\left(x_{1}^{\mathrm{gt}},y_{1}^{\mathrm{gt}}\right)$ from the predicted and ground truth boxes, along with their bottom‐right corners $\left(x_{2}^{\mathrm{prd}},y_{2}^{\mathrm{prd}}\right)$ and $\left(x_{2}^{\mathrm{gt}},y_{2}^{\mathrm{gt}}\right)$. We then compute the sum of squared Euclidean distances between corresponding corner points, denoted as $d_{1}^{2}={\left(x_{1}^{\mathrm{prd}}-x_{1}^{\mathrm{gt}}\right)}^{2}+{\left(y_{1}^{\mathrm{prd}}-y_{1}^{\mathrm{gt}}\right)}^{2}$ and $d_{2}^{2}={\left(x_{2}^{\mathrm{prd}}-x_{2}^{\mathrm{gt}}\right)}^{2}+{\left(y_{2}^{\mathrm{prd}}-y_{2}^{\mathrm{gt}}\right)}^{2}$. The MDPIoU metric is finally calculated using the following formula:

(12) $$\text{MPDIoU}=\mathrm{IoU}-\frac{d_{1}^{2}+d_{2}^{2}}{w^{2}+h^{2}}$$

where IoU represents the conventional intersection‐over‐union ratio, while w and h denote the width and height of the input image, respectively. The loss function is formulated as $L_{\text{MPDIoU}}=1-\text{MDPIoU}$, which enables direct optimization of keypoint distances to achieve efficient bounding box regression.

Figure 2d illustrated two examples of bounding box regression results: the green box indicated the true bounding box, while the red box denoted the predicted bounding box. In both examples, traditional loss functions such as Generalized Intersection over Union (GloU), Distance Intersection over Union (DloU), Complete Intersection over Union (CloU), and Efficient Intersection over Union (EloU) yielded identical loss values (Ma and Xu 2023). In contrast, the MPDIoU method produced distinct loss values, underscoring its effectiveness in improving the accuracy of bounding box predictions.

#### 2.2.5 ByteTrack Tracking Head

The ByteTrack algorithm, as elucidated by Ma and Xu (2023), is recognized for its efficiency, simplicity, and robustness in multi‐target tracking, particularly noted for its accuracy. A key challenge it addresses is the loss of targets during occlusion, which it mitigates by associating nearly all detection boxes. As illustrated in Figure 2e, the tracking process began with the ByteTrack tracking head algorithm processing the current frame through the AnimalYOLO target detector to identify a series of potential target boxes. These identified boxes were then subjected to prediction using the Kalman filtering technique. The Kalman filter estimates the target's position and velocity in the current frame by propagating the previous frame's trajectory state (containing position, velocity, and other motion parameters). The prediction step computes the a priori state vector x^k and corresponding covariance matrix P^k through the following system equations:

(13) x^k=Fkxk−1+Bkuk

(14) P^k=FkPk−1FkT+Qk

where $F_{k}$ is the state transfer matrix, $B_{k}$ is the control input matrix, $u_{k}$ is the control vector, and $Q_{k}$ is the process noise covariance matrix. The predicted bounding box coordinates are extracted by the state vector x^k for subsequent data correlation.

To establish data correlation, the Hungarian Algorithm was subsequently applied, pairing detection boxes with historical trajectories (Zhang et al. 2022). High‐scoring target boxes were directly matched with established trajectories, whereas lower‐scoring boxes were aligned with previously unmatched trajectories, a critical step for recognizing occluded targets.

### 2.3 Quantification of the Slow Loris's Movement

**FIGURE 3:**
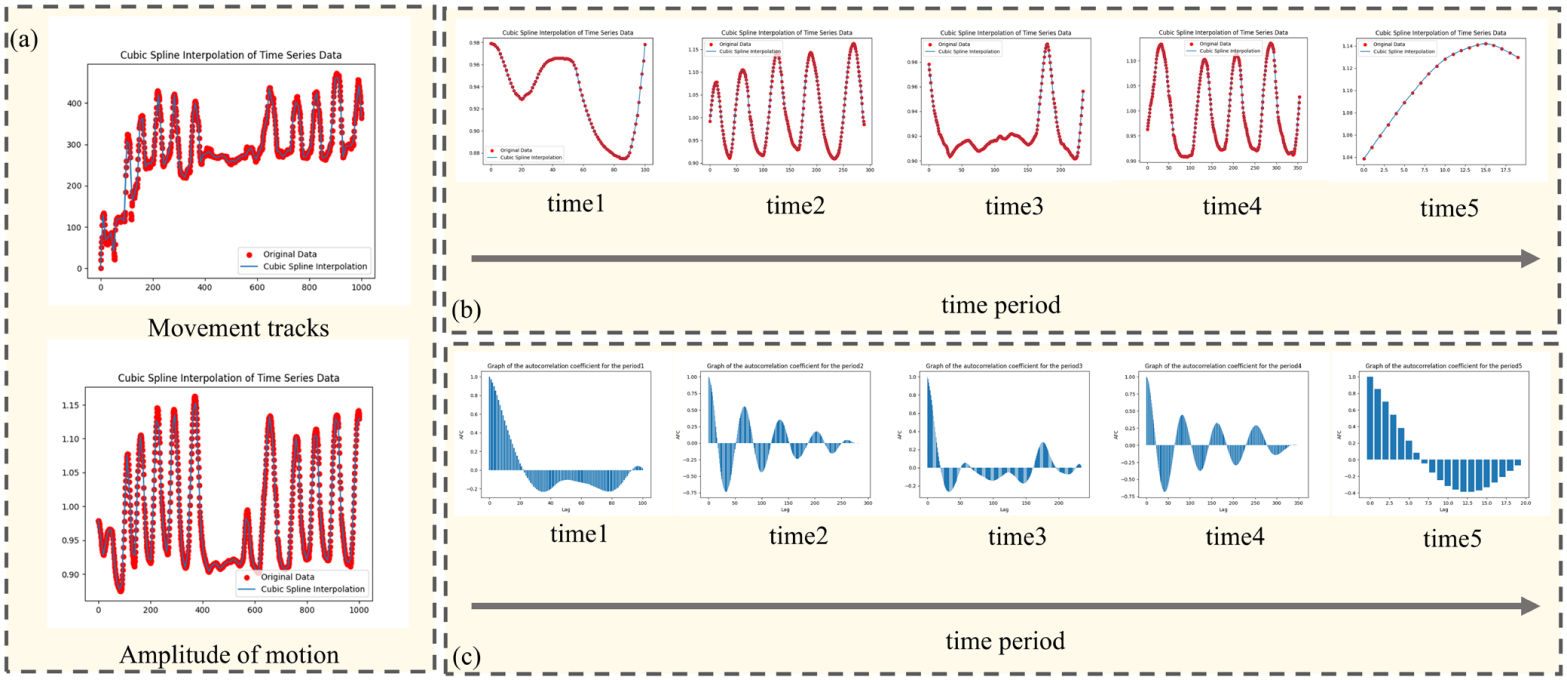
Example of small displacement stereotypical behavior identification results. (a) Comparison of three‐sample interpolation of motion trajectory and amplitude, (b) examples of data segmentation results, and (c) the autocorrelation function plots for each data segment.

In a constrained environment, the changes in the center‐of‐mass coordinates of the slow loris were not significant. However, the detection boxes accurately conformed to the contours of the individuals in each frame, leading to variations in their size and shape due to the lorises' movement. Given the cyclic nature of the slow loris's stereotypic behavior, these variations in the shape of the detection boxes during each cycle exhibited similarities. To quantify the movement amplitude, this study employs the aspect ratio coefficient of the detection boxes as an index. After calculating the aspect ratio coefficients for each frame, a cubic spline interpolation algorithm was applied to smooth the data, constructing a continuous curve representing the change in movement amplitude. Spline interpolation plotting was performed three times for both movement trajectory and movement amplitude. Due to the small displacement characteristic of the stereotypical behavior of caged slow lorises, the periodicity of the plotted trajectory curves was not prominent. As shown in Figure 3a, the movement trajectory graph exhibited significant data overlap and shift, making the trajectory curve less discernible. In contrast, the amplitude of movement graph displayed much clearer periodicity curves, providing a more expressive representation of the phenomenon. Overall, quantifying movement amplitude proves to be more suitable for studying the stereotypical behavior of slow lorises with small displacements.

#### 2.3.1 Segmentation of Data Series in Stereotypical Behavior

The stereotypical behavior of captive slow lorises does not follow a continuous, uninterrupted pattern. Instead, it is intermittent, with non‐stereotypical behaviors showing movement curves that typically lack fluctuations (Figure 3a). This leads to distinctly different patterns of change in the data, and directly plotting the autocorrelation function for the entire dataset would significantly compromise its accuracy. Therefore, we employ the dichotomous outlier point detection method (SPSSPRO, n.d.) to effectively segment the data series. Figure 3b illustrates the examples of the data segmentation process, emphasizing the identification of outlier points within the time series. This method efficiently uncovers turning points and changing trends, which are essential for pinpointing data segments that exhibit distinct shifts or variations in the movement amplitude. By identifying these critical points, the approach enhances the analysis of the slow loris's movement patterns by discerning segments with clear changes or differing trends.

#### 2.3.2 Periodicity Judgment

The segmented curves illustrating the variation in movement amplitude clearly demonstrate the remarkable periodicity of these curves during periods when the stereotypical behavior is prominently displayed (Figure 3b). Each cycle commences when the slow loris performs a specific action and concludes with the repetition of a similar action, at which point the aspect ratio coefficients of the actions before and after exhibit a high degree of consistency. Throughout the cycle, the values of these coefficients undergo a series of changes due to the movement characteristics. Consequently, we designate the point of consistency in the aspect ratio coefficients as the demarcation point for the cyclic changes in the slow loris's behavior, thereby ensuring an accurate division of the cycle.

In time series analysis, the autocorrelation coefficient (ACF) is extensively utilized to identify the periodicity, as it reflects the self‐similarity between a signal and itself over various time delays. The equation is as follows.

(15) $$\mathrm{ACF}\left(k\right)=\sum_{i=k+1}^{n}\frac{\left(y_{i}-\bar{y}\right)\left(y_{i-k}-\bar{y}\right)}{\sum_{i=1}^{n}{\left(y_{i}-\bar{y}\right)}^{2}}$$

where *k* denotes the lag factor, $y_{i}$ denotes the movement amplitude of the slow loris in the *i*th frame, $\bar{y}$ denotes the average of the movement amplitudes of all the individuals. Figure 3c illustrates the autocorrelation function plots for various data segments following division. The periodicity of the data is typically manifested as a recurring pattern in these plots.

We employ the Fast Fourier Transform (FFT) to determine specific cycle sizes of stereotypical behaviors. The FFT is a signal processing technique that converts a signal from the time domain to the frequency domain. In this transformation, the sine wave frequency with the largest amplitude typically represents the primary periodic feature, which influences the periodicity and overall wave structure of the signal.

### 2.4 Experimental Environment

The experimental setup utilizes an Ubuntu (64‐bit) operating system running on a 12‐core Intel(R) Xeon(R) Platinum 8255C CPU@2.50GHz processor, with an RTX 3080 GPU. The PyTorch open‐source deep learning framework serves as the development environment, with Python 3.8, a CUDA version of 11.1, and 43GB of computer memory.

### 2.5 Evaluation Indicators

#### 2.5.1 Evaluation of Target Detection

We used three metrics to assess the detection of individual slow lorises: Precision, mean Average Precision (mAP), and Recall. Table 1 provides the definitions of the parameters used in the formulas.

**TABLE 1:** Parameters of evaluation indicators.

Confusion matrix		Predicted results	
		Positive	Negative
Expected results	Positive	TP	FN
	Negative	FP	TN

TP represents the number of samples correctly predicted as positive by the model, FP refers to the number of samples incorrectly predicted as positive by the model, FN is the number of samples incorrectly predicted as negative by the model, and TN indicates the number of samples correctly predicted as negative by the model.

Precision indicates the probability that a sample predicted to be positive will actually be positive. The formula is as follows:

(16) $$\text{Precision}=\frac{\mathrm{TP}}{\mathrm{TP}+\mathrm{FP}}$$

AP denotes the integral from the P‐index to the R‐index. mAP refers to the average of the AP values for all categories. The formula is as follows:

(17) $$\mathrm{AP}=\int_{0}^{1}P\left(R\right)\mathrm{dR}$$

(18) $$\mathrm{mAP}=\frac{1}{K}\sum_{i=1}^{K}AP_{i}$$

Recall represents the proportion of individuals that the model correctly judges to be in the positive category out of all individuals that are actually in the positive category. The formula is as follows:

(19) $$\text{Recall}=\frac{\mathrm{TP}}{\mathrm{TP}+\mathrm{FN}}$$

#### 2.5.2 Evaluation of Object Tracking

We employed three metrics—Identity F1 Score (IDF1), Recall, and Multiple Object Tracking Accuracy (MOTA)—to evaluate the tracking performance of individual slow lorises. IDF1 is the ratio of the number of correctly identified detections to the sum of the average true and calculated detections, calculated as follows:

(20) $$\mathrm{IDF}1=\frac{2}{\frac{1}{\mathrm{IDP}}+\frac{1}{\mathrm{IDR}}}=\frac{2\text{IDTP}}{2\text{IDTP}+\text{IDFP}+\text{IDFN}}$$

where identity precision (IDP) is the calculated detection score for correct identification and identity recall (IDR) is the score for correctly identifying the ground truth detection, their specific formulas are as follows:

(21) $$\mathrm{IDP}=\frac{\text{IDTP}}{\text{IDTP}+\text{IDFP}}$$

(22) $$\mathrm{IDR}=\frac{\text{IDTP}}{\text{IDTP}+\text{IDFN}}$$

Recall is a metric used to evaluate target detection, defined as the ratio of correctly matched detection targets to the total number of targets specified by the ground truth. This calculation is analogous to how Recall is determined in target detection tasks. Meanwhile, the MOTA metric provides a comprehensive assessment of target tracking performance by considering several factors, including false detections, missed detections, and identity switches. This evaluation is conducted using the following formulae:

(23) $$\text{MOTA}=1-\frac{\mathrm{FN}+\mathrm{FP}+\text{IDSW}}{\mathrm{GT}}$$

#### 2.5.3 Evaluation Method for Stereotypical Behavior Recognition Algorithms

We employ the Darklabel tool to conduct fine‐grained annotations on the slow loris tracking data, which will serve as input for the recognition algorithm. Additionally, we manually measure the cycle length and duration of stereotypical behavior in slow lorises to obtain ground truth values (Benchmark) for evaluating our stereotypical behavior algorithm. Specifically, we manually segment videos exhibiting stereotypical behavior to identify the time intervals and durations of these behaviors, count the number of stereotypical behavior events, and define the cycle as the ratio of the total duration of stereotypical behavior to the corresponding number of events.

To assess the algorithm's performance, we calculate the prediction errors for both the average cycle time and the duration of stereotypical behavior. The cycle time prediction error is determined by computing the mean absolute error between the algorithm's predicted cycle times across all videos and the ground truth values. Similarly, the behavior duration prediction error is derived from the mean absolute error between the algorithm's predicted durations and the actual measured values.

## 3 Results

### 3.1 Results of Ablation Studies and Comparative Experiments for Object Detection

**FIGURE 4:**
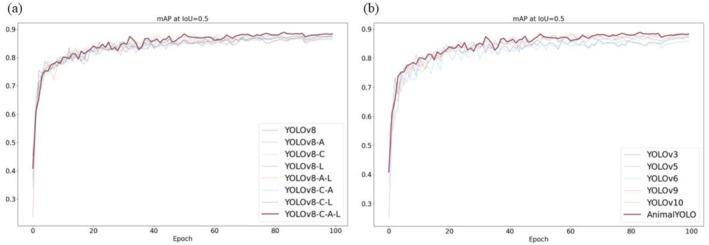
The results of the ablation and comparison experiment across various target detection methods. (a) illustrates the evolution of mAP throughout the ablation study iterations, while (b) presents the corresponding results from the comparison experiments, contrasting the mAP progression across benchmark models.

To rigorously evaluate the performance of the optimization algorithms employed in this study, a series of meticulously designed ablation experiments were conducted. These experiments aimed to ascertain the contribution of each component to the overall performance by systematically removing or replacing key elements of the algorithm. Within the context of these experiments, the symbol “√” indicates the application of a module within the network, where “C” represents the SWS module, “A” denotes the MCA module, and “L” signifies the MPD loss function. The accuracy percentages resulting from these ablation experiments are presented in Table 2. These findings, along with data illustrating the increase in the model's mean Average Precision (mAP) with the number of training rounds, as shown in the left panel of Figure 4, indicate that the configuration YOLOv8‐C‐A‐L, referred to as AnimalYOLO, is optimal for detection, achieving a mAP value of 88.9%.

**TABLE 2:** Results of ablation experiments (%). The final model (YOLOv8‐C‐A‐L) achieved an mAP of 88.9%, which represents an improvement of 2.1 percentage points over the baseline, as indicated by the bolded results in the table.

Models	C	A	L	Precision	Recall	mAP
YOLOv8s				0.958	0.786	0.868
YOLOv8‐C	√			0.979	0.798	0.875
YOLOv8‐A		√		0.959	0.791	0.877
YOLOv8‐L			√	0.969	0.776	0.872
YOLOv8‐C‐A	√	√		0.972	0.794	0.884
YOLOv8‐C‐L	√		√	0.968	0.800	0.876
YOLOv8‐A‐L		√	√	0.949	0.799	0.883
YOLOv8‐C‐A‐L	√	√	√	0.967	0.803	**0.889**

*Note:* C, SWS convolution module; A, multi‐dimension cooperative attention; L, MPDIoU algorithm.

In the AnimalYOLO validation set, the images, which were derived from video frame extraction, exhibited a high degree of similarity. Despite this similarity, the model effectively detected subtle movements of the slow loris. As a result, the detection boxes more accurately aligned with the edges of the slow loris, as demonstrated in Figure S4.

To ensure a comprehensive and fair evaluation, we introduced several existing frameworks to conduct a series of comparative experiments. The results demonstrated the superiority of the AnimalYOLO algorithmic framework across various target detection methods in terms of efficiency and performance (Table 3, Figure 4).

**TABLE 3:** Comparison results of different target detection methods. The final model (YOLOv8‐C‐A‐L) achieved a mean Average Precision (mAP) of 88.9%, which represents an improvement of approximately 1% over all other benchmarked models, as indicated by the bolded results in the table.

Models	Precision	Recall	mAP
YOLOv3	0.974	0.779	0.871
YOLOv5	0.942	0.801	0.879
YOLOv6	0.954	0.782	0.857
YOLOv9	0.944	0.794	0.874
YOLOv10	0.966	0.790	0.878
AnimalYOLO	0.967	0.803	**0.889**

### 3.2 Object Tracking Performance Evaluation Results

The IDF1 metric reached a maximum value of 88.4%, while the MOTA metric achieved a peak value of 95.6% in the evaluated videos (Table 4). These results indicated that our multi‐target tracking network performed exceptionally well in certain video scenes, particularly those filmed with a red flashlight for auxiliary illumination. However, in videos with complex environmental backgrounds and gray light, such as Video_24, Video_25, Video_28, and Video_35, both the IDF1 and MOTA metrics were comparatively lower (Figure S5).

**TABLE 4:** Summary statistics in evaluating the performance of the slow loris tracker in various video files.

Video file	IDF1	Recall	MOTA
Video_6	55.9%	100.0%	91.6%
Video_7	88.4%	100.0%	91.7%
Video_9	64.3%	61.7%	60.5%
Video_11	62.4%	59.5%	59.3%
Video_23	65.1%	100.0%	95.6%
Video_24	48.7%	34.5%	33.9%
Video_25	49.7%	37.7%	37.2%
Video_27	82.4%	99.8%	57.6%
Video_28	40.6%	23.9%	22.5%
Video_35	50.0%	33.4%	33.3%
Video_37	54.4%	44.7%	44.4%
Video_39	63.9%	79.0%	77.9%
Video_44	54.5%	72.3%	71.4%
Overall	59.2%	58.3%	55.3%

### 3.3 Assessment of Stereotypical Behavioral Judgments

The results indicated a prediction error of 0.33 s for the video cycle time and 0.76 s for the duration of stereotypical behaviors, demonstrating the feasibility and superiority of our proposed method for the automatic detection. Table 5 provides videos of slow lorises exhibiting stereotypical behaviors in both single‐target and multiple‐target scenarios, aiding in the identification of animals displaying such behaviors in multi‐target settings. It is important to note that “None” signifies that the slow loris in the video did not exhibit stereotypical behaviors.

**TABLE 5:** The predicted mean cycle time and duration for determining stereotypic behavior of slow lorises in various video files.

Name	ID	Cycle time		Duration	
		Benchmark	Predicted	Benchmark	Predicted
Video_6	1	1.37	1.68	12.45	11.72
Video_7	1	2.58	2.19	4.57	6.58
	2	None	None	None	None
Video_9	1	1.81	1.69	8.00	8.46
	2	None	None	None	None
	3	None	None	None	None
	4	None	None	None	None
	5	None	None	None	None
	6	None	None	None	None
Video_11	1	3.03	2.57	17.81	17.98
	2	None	None	None	None
	3	None	None	None	None
Video_23	1	None	None	None	None
	2	1.34	1.37	3.92	5.43
	3	None	None	None	None
Video_24	1	None	None	None	None
	2	2.04	1.96	10.40	11.74
	3	None	None	None	None
	4	None	None	None	None
	5	None	None	None	None
	6	None	None	None	None
	7	None	None	None	None
	8	None	None	None	None
	9	None	None	None	None
	10	None	None	None	None
Video_25	1	1.68	1.66	8.21	8.92
	2	None	None	None	None
	3	None	None	None	None
Video_27	1	1.38	1.37	13.16	12.18
	2	None	None	None	None
Video_28	1	1.96	1.63	6.89	6.49
Video_35	1	2.33	1.67	12.62	11.72
	2	None	None	None	None
Video_37	1	0.93	0.90	14.37	14.50
	2	None	None	None	None
	3	None	None	None	None
Video_39	1	None	None	None	None
	2	None	None	None	None
	3	1.32	0.67	5.87	5.92
Video_44	1	4.23	3.49	17.26	17.45
	2	4.24	3.71	18.60	18.45
	3	1.80	1.28	14.37	12.77

### 3.4 Applicability in Other Species

We extended the analysis to incorporate different species exhibiting similar behaviors (Figure S6). The AnimalYOLO target detection model achieved a higher mAP value (0.973) compared to YOLOv8 (0.970), and the model's effectiveness in tracking other animals got an average IDF1 score of 79.3%, which exceeded the performance for the slow loris (Table 6). However, the Multiple Object Tracking Accuracy (MOTA) was slightly lower than that for the slow loris. The mean prediction errors for cycle period and duration were 0.10 s and 0.32 s, respectively, both lower than the error values for the slow loris (Table 7).

**TABLE 6:** AnimalYOLO‐Bytetrack's tracking results for other animals.

Video name	IDF1	Recall	MOTA
Video_1 (chimpanzee)	79.7%	73.8%	64.1%
Video_2 (brown bear)	39.7%	39.8%	33.7%
Video_3 (domestic cats)	70.7%	42.4%	30.1%
Video_4 (brown bear)	99.0%	100.0%	98.0%
Video_5 (polar bear)	22.9%	11.7%	10.4%
Video_6 (brown bear)	94.4%	98.8%	89.9%
Video_8 (domestic cats)	95.0%	99.7%	91.7%
Video_9 (rhesus macaque)	90.0%	56.0%	30.2%
Overall	79.3%	64.5%	52.9%

**TABLE 7:** The predicted mean cycle time and duration for determining stereotypic behavior of other animals in various video files.

Name	ID	Cycle time		Duration	
		Benchmark	Predicted	Benchmark	Predicted
Video_1	1	0.64	0.60	18.97	19.23
	2	None	None	None	None
	3	None	None	None	None
	4	None	None	None	None
Video_2	1	0.73	0.81	11.60	11.40
	2	None	None	None	None
	3	None	None	None	None
Video_3	1	0.90	0.44	6.33	7.00
Video_4	1	0.50	0.46	9.25	9.75
	2	None	None	None	None
Video_5	1	0.62	0.56	4.47	4.50
Video_6	1	1.52	1.39	4.00	4.17
	2	None	None	None	None
Video_8	1	0.96	0.95	7.13	6.53
Video_9	1	1.18	1.19	8.23	8.33

## 4 Discussion

### 4.1 Methodological Advances and Contributions

This study successfully addresses the critical gap in automated monitoring of small‐displacement stereotypical behaviors in multi‐animal captive settings. We developed AnimalYOLO‐Bytetrack, an integrated framework combining enhanced multi‐target tracking, novel movement quantification via bounding box aspect ratio changes, and robust periodicity analysis. This approach achieved relatively high accuracy in detecting subtle, repetitive behaviors like head shaking and turning in captive slow lorises, with a mean Average Precision (mAP) of 88.9%, detection precision of 96.7%, recall of 80.3%, and low prediction errors for cycle time (0.33 s) and behavior duration (0.76 s). Crucially, our method overcomes several key limitations of prior approaches. Traditional methods relying on centroid trajectory analysis (Yin et al. 2024) are ineffective for minimal‐movement stereotypies where positional shifts are negligible (e.g., head shaking within a cage). By innovatively using the periodic variation in the aspect ratio of detection boxes tightly fitted to the animal's contour (enabled by MPDIoU loss), we established a sensitive proxy for subtle movement amplitude changes, proving significantly more suitable than trajectory plotting for identifying small‐displacement periodicity. Unlike frame‐based posture classification (Mathis et al. 2018) or accelerometer data processing (Hathaway et al. 2023), our time‐series analysis of movement amplitude directly captures the temporal repetitiveness inherent in stereotypies. The segmentation‐ACF‐FFT pipeline robustly identifies specific behavioral episodes and their cycle characteristics. In addition, the integration of MCA and SWS modules within AnimalYOLO enhanced feature discrimination in cluttered rescue center backgrounds (stacked cages), while ByteTrack's strategy of associating low‐confidence detections improved tracking continuity during occlusions common in multi‐individual settings (Ou and Shen 2024; You et al. 2023; Zhang et al. 2025).

### 4.2 Performance Analysis and Generalizability

The framework demonstrated strong performance in tracking and behavior prediction for slow lorises, particularly under favorable conditions like red‐light auxiliary illumination. However, performance declined in videos with complex backgrounds and gray light. This highlights the persistent challenge of target detection and ID maintenance under suboptimal lighting and high background clutter, directly impacting subsequent behavior quantification accuracy. Notably, the method showed promising generalizability to other species exhibiting small‐displacement stereotypies (bears, primates, domestic cats). While the average IDF1 score (79.3%) exceeded that for slow lorises (59.2%), the overall MOTA (52.9%) was slightly lower, and prediction errors for cycle time (0.10 s) and duration (0.32 s) were reduced. This discrepancy likely stems from dataset composition, animal size, and detection challenges. Specifically, videos of other species often featured single animals in less cluttered enclosures, simplifying detection and tracking compared to the complex, multi‐loris cage environments typical of our primary dataset. Larger animals (e.g., bears) usually exhibited more pronounced displacement movements during their stereotypies compared to the subtle motions of slow lorises. This potentially facilitated more accurate bounding box fitting and movement amplitude quantification, contributing to lower prediction errors. In addition, the necessity to detect and track a wider variety of species and behaviors increased the likelihood of misdetections (FP) and omissions (FN), negatively impacting the comprehensive MOTA metric, which aggregates these errors (Wojke et al. 2017).

### 4.3 Limittations and Future Directions

This study acknowledges several limitations. Acquiring sufficient video data was challenging due to the exclusion of severely impaired slow lorises (e.g., those with mobility issues from electrocution injuries), restricting dataset size and diversity. Furthermore, the spatially constrained captive environment at the rescue center, characterized by vertically stacked cages, generated complex visual scenes that significantly degraded multi‐target tracking performance, highlighting the practical difficulties of deploying automated monitoring in resource‐limited rescue settings compared to controlled exhibits. The lack of detailed individual metadata (age, sex, health history, time in captivity) for most slow lorises impedes deeper analysis of factors influencing stereotypy development. Nevertheless, our observation that group‐housed lorises (Video_41) showed no stereotypy, contrasting with a solitary individual in a shared cage (Video_8), aligns with previous studies suggesting social housing may mitigate stress and abnormal behaviors (Alejandro et al. 2021). Crucially, while our method effectively quantifies motor parameters of stereotypies (timing, duration, periodicity), it does not capture essential non‐motor indicators of welfare states or behavioral triggers (e.g., pupillary response, piloerection, specific vocalizations), which remain vital for a holistic understanding and are typically assessed through direct keeper or veterinary observation.

Despite these limitations, the AnimalYOLO‐Bytetrack framework offers significant practical advantages over manual methods, enabling continuous monitoring and automatic logging of movement amplitude trends to precisely identify the onset, duration, and periodicity of stereotypical episodes. This functionality provides animal managers with a valuable tool for visualizing behavioral data and has the potential to generate real‐time alerts upon stereotypy detection, facilitating prompt interventions such as environmental enrichment or adjustments to social groupings to alleviate animal stress (Moore et al. 2015). Looking ahead, future research should prioritize developing unified algorithms capable of detecting both small and large displacement stereotypies, potentially leveraging inter‐frame similarity analysis (e.g., Siamese Networks, RepNet) (Chicco 2021; Dwibedi et al. 2020) to identify repetitive patterns irrespective of movement magnitude. Further enhancements should focus on integrating multimodal sensing (e.g., audio, thermal imaging) to capture non‐motor welfare indicators, improving robustness under extreme lighting and background conditions, and applying the tool to longitudinal datasets to investigate links between stereotypy metrics, management changes, and broader health outcomes in captive populations.

## 5 Conclusion

This study presents a novel method for recognizing stereotypical behaviors characterized by small displacements in slow lorises and other animals, employing computer vision‐based and deep learning algorithms. By implementing an efficient multi‐target tracking algorithm, we have innovatively applied a small‐displacement stereotypical behavior analysis method that relies on periodic variations in movement amplitude. The approach provides a valuable tool for enhancing animal welfare monitoring in rescue centers and zoos, facilitating timely interventions and contributing to improved management strategies for species susceptible to captivity‐induced stress. Future work integrating broader behavior detection and multimodal sensing holds promise for even more comprehensive welfare assessment.

## Author Contributions

**Ziqi Yang:** conceptualization (equal), data curation (equal), formal analysis (equal), funding acquisition (equal), investigation (equal), methodology (equal), project administration (equal), resources (equal), software (equal), supervision (equal), validation (equal), visualization (equal), writing – original draft (equal), writing – review and editing (equal). **Yating Du:** conceptualization (equal), data curation (equal), formal analysis (equal), funding acquisition (equal), investigation (equal), methodology (equal), project administration (equal), resources (equal), supervision (equal), writing – review and editing (equal). **Peifeng Li:** conceptualization (equal), formal analysis (equal), investigation (equal), methodology (equal), project administration (equal), software (equal), supervision (equal), validation (equal), visualization (equal). **Tingting Leng:** conceptualization (equal), formal analysis (equal), investigation (equal), project administration (equal), software (equal), validation (equal), visualization (equal). **Shaoyun Ding:** conceptualization (equal), formal analysis (equal), investigation (equal), methodology (equal), software (equal), visualization (equal). **Yuxin Zhang:** software (equal), validation (equal), visualization (equal). **Zhenzhen Lin:** software (equal), validation (equal), visualization (equal). **Yilin Yang:** software (equal), validation (equal), visualization (equal). **Yumai Fan:** data curation (equal), resources (equal). **Yan Zhang:** data curation (equal), resources (equal). **Changjun Zeng:** data curation (equal), resources (equal). **Meng Xie:** data curation (equal), resources (equal). **Qingyong Ni:** conceptualization (equal), data curation (equal), formal analysis (equal), funding acquisition (equal), resources (equal), supervision (equal), writing – review and editing (equal).

## Conflicts of Interest

The authors declare no conflicts of interest.

## Supporting information


**Figure S1:** Example of video data from captive slow lorises. The dataset contains both normal and stereotyped behaviors.
**Figure S2:** Examples of image enhancement.
**Figure S3:** Video examples of the stereotypical behavior.
**Figure S4:** AnimalYOLO partial validation set.
**Figure S5:** Example of tracking results for daytime conditions as well as for frames illuminated by two different light sources.
**Figure S6:** Examples of animal tracking results in some other species with small displacement stereotypical behaviors.

## Data Availability

The data that support the findings of this study are openly available in figshare at http://doi.org/10.6084/m9.figshare.28368149.v1, reference number 28368149.v1. The code that supports the findings of this study is openly available in GitHub at https://github.com/yangziqi2003/AnimalYOLO‐ByteTrack‐and‐data/tree/master.
