# Supplementary material for: Identifying Small‐Displacement Stereotypical Behavior of Captive Slow Lorises Based on AnimalYOLO‐Bytetrack Network
Source: Ecol Evol. 2025 Oct 10;15(10):e72304. doi: 10.1002/ece3.72304 (PMC12511953; doi:10.1002/ece3.72304)
Supplement: Supplementary file 1 — Figure S1: Example of video data from captive slow lorises. The dataset contains both normal and stereotyped behaviors. Figure S2: Examples of image enhancement. Figure S3: Video examples of the stereotypical behavior. Figure S4: AnimalYOLO partial validation set. Figure S5: Example of tracking results for daytime conditions as well as for frames illuminated by two different light sources. Figure S6: Examples of animal tracking results in some other species with small displacement stereotypical behaviors. [file ECE3-15-e72304-s001.docx]

Supplemental files


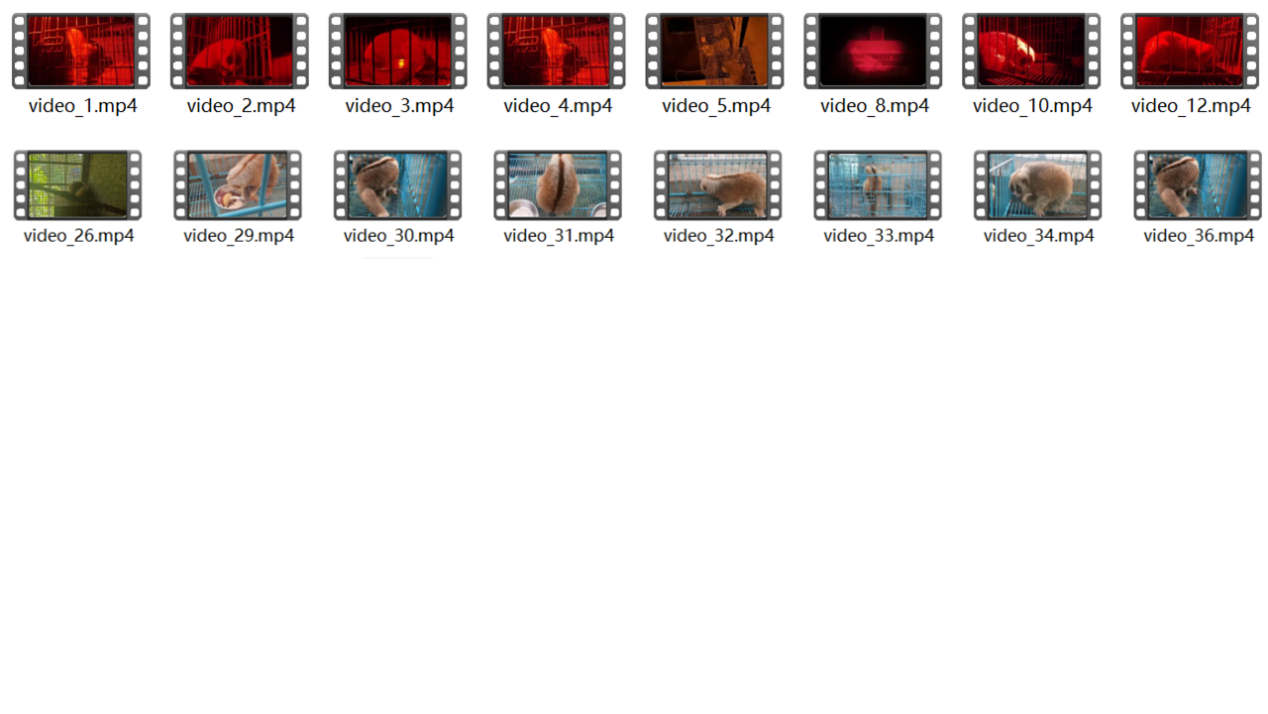


**Figure S1. Example of video data from captive slow lorises. The dataset contains both normal and stereotyped behaviours.**


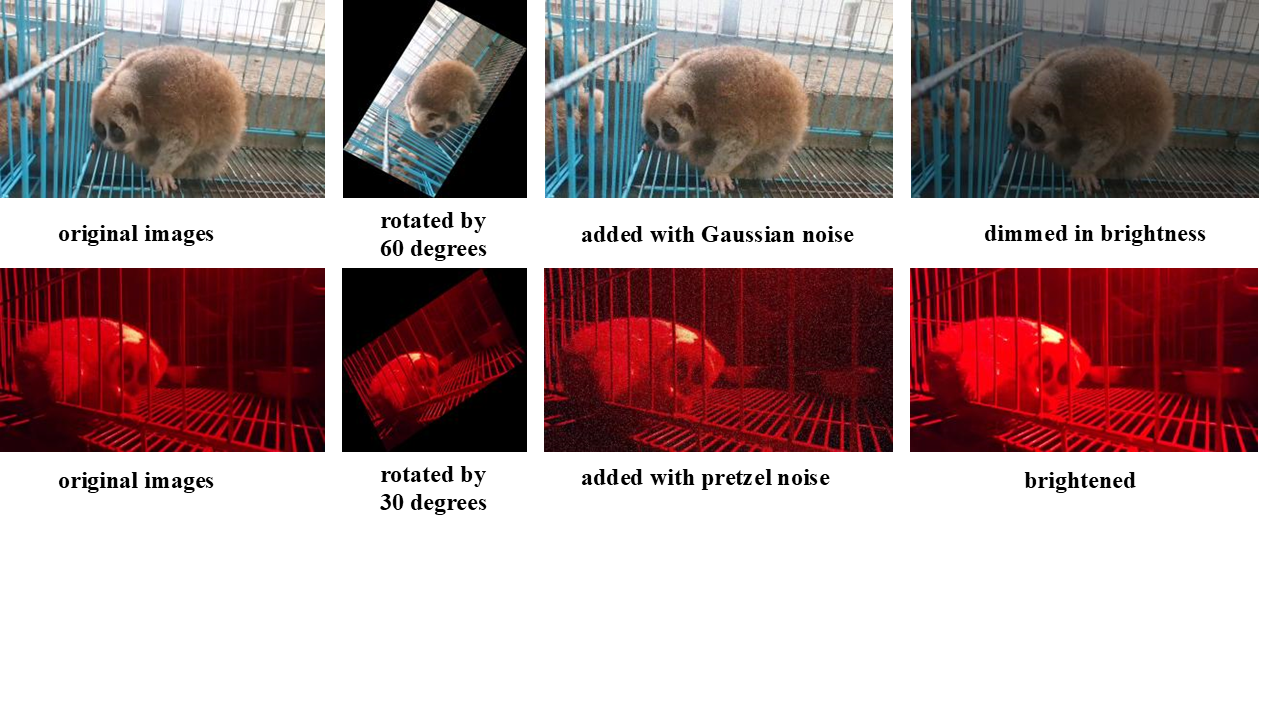


**Figure S2. Examples of image enhancement.**


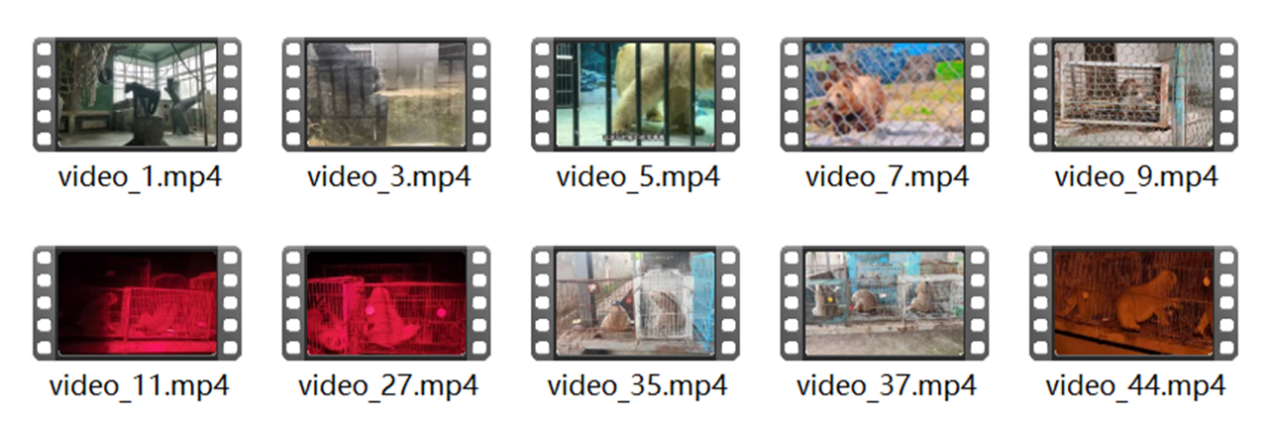


**Figure S3. Video examples of the stereotypical behaviour.**


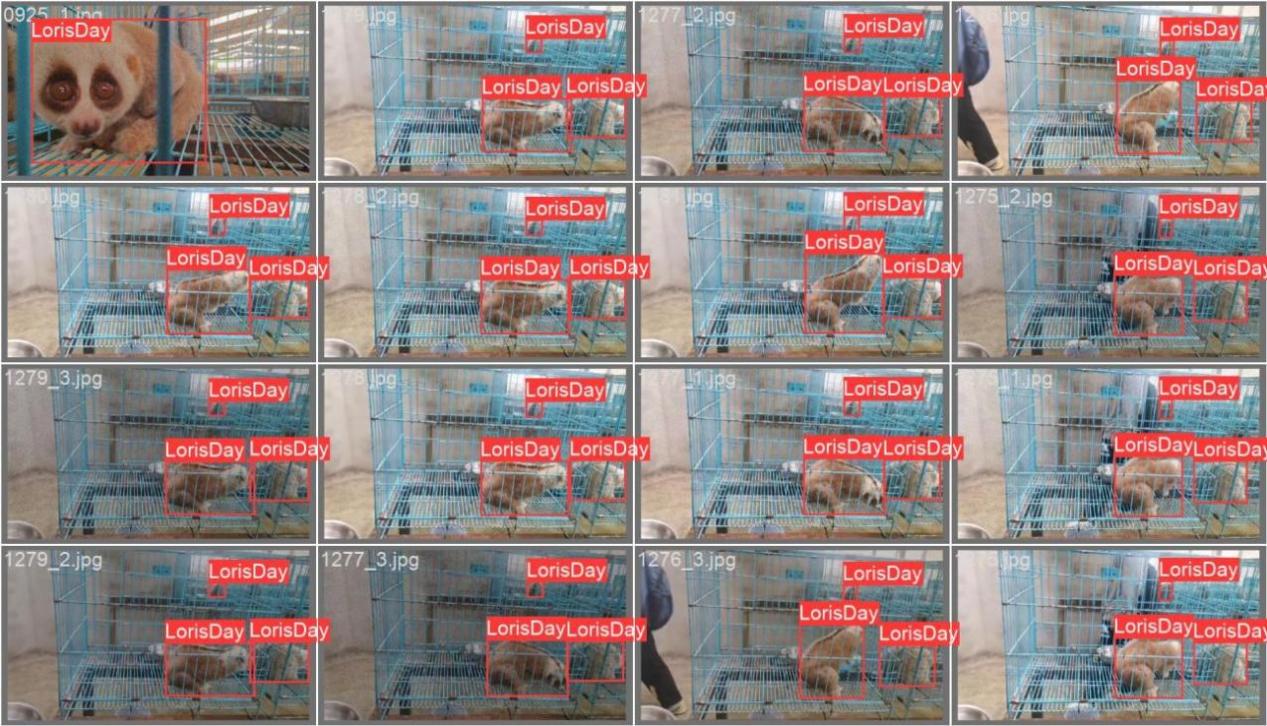


**Figure S4. AnimalYOLO partial validation set**


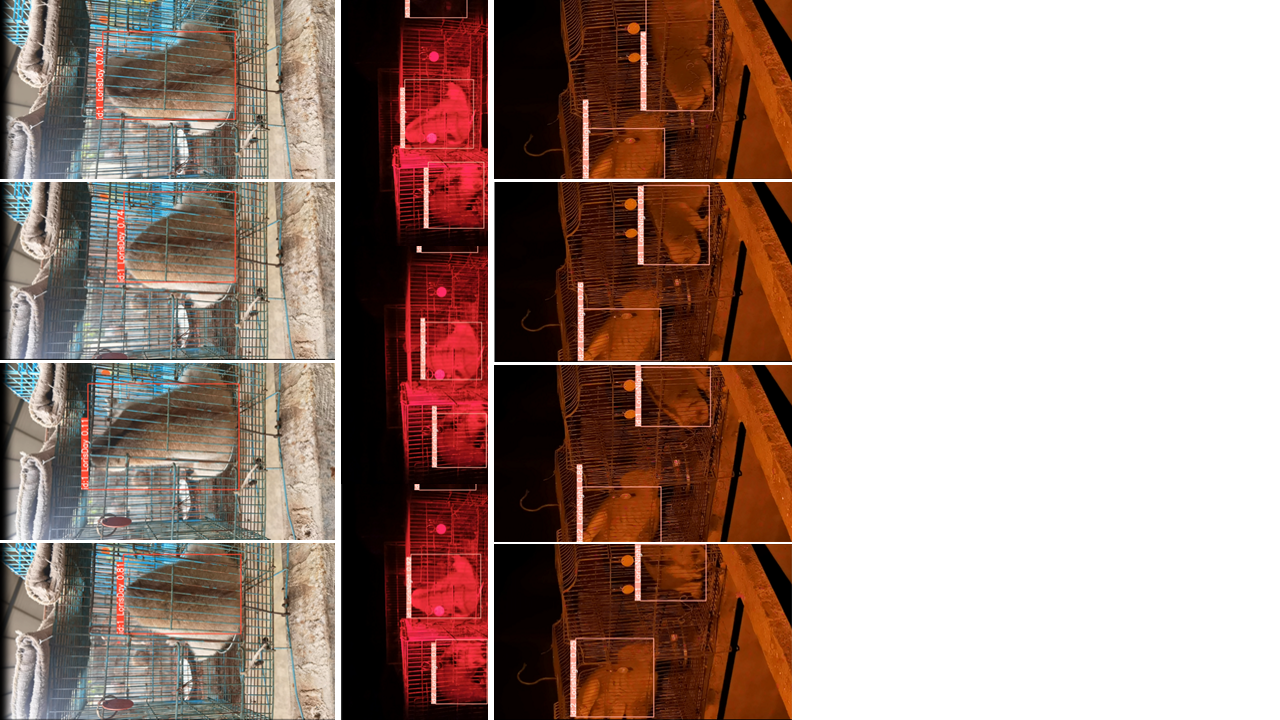


**Figure S5. Example of tracking results for daytime conditions as well as for frames illuminated by two different light sources.**

**
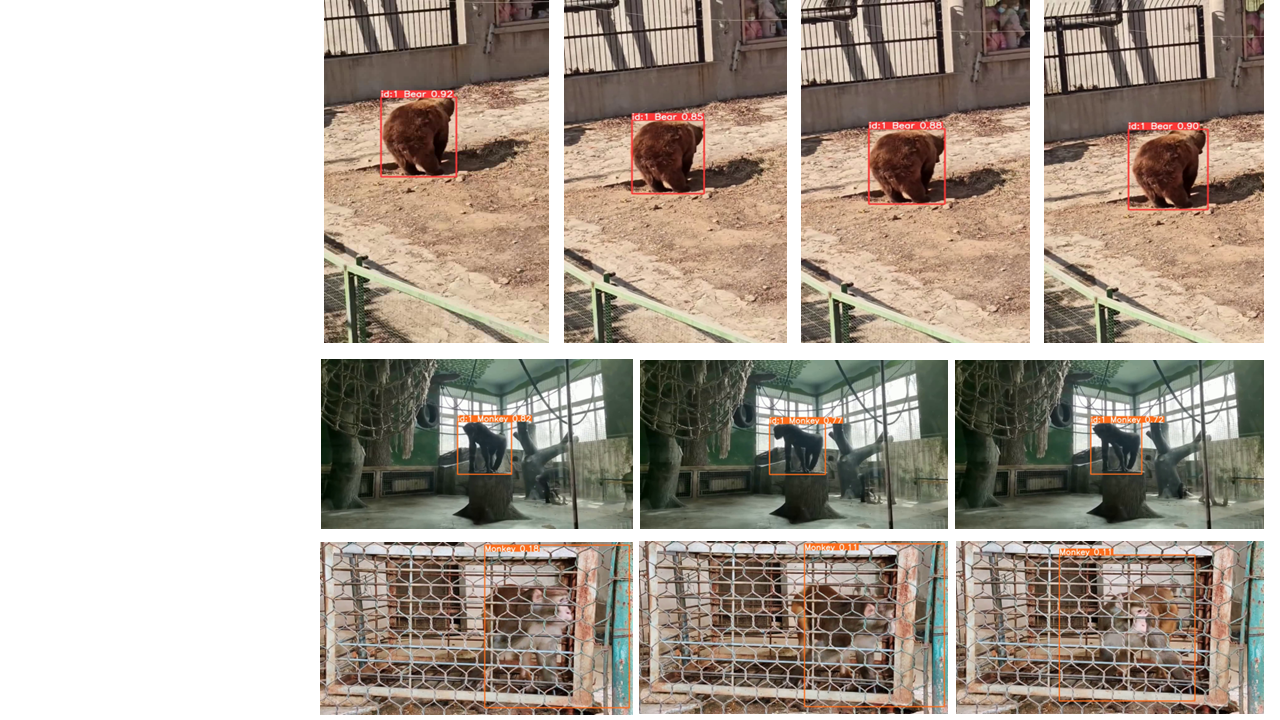
**

**Figure S6 . Examples of animal tracking results in some other species with small displacement stereotypical behaviours**
